# Impact of adenomyosis on pregnancy outcomes following frozen embryo transfer: development and validation of a single-center clinical predictive model

**DOI:** 10.3389/fendo.2026.1770158

**Published:** 2026-03-05

**Authors:** Huan Chen, Linghui Weng, Yichen Chen, Liping Chen, Jue Zhu, Jing Zhang

**Affiliations:** 1 Ningbo University, Ningbo, Zhejiang, China; 2 Department of Gynecology, Women and Children’s Hospital of Ningbo University, Ningbo, Zhejiang, China; 3 Central Laboratory, Women and Children’s Hospital of Ningbo University, Ningbo, Zhejiang, China

**Keywords:** adenomyosis, clinical pregnancy, frozen embryo transfer, live birth, prediction model

## Abstract

**Objective:**

To identify factors associated with clinical pregnancy and live birth in frozen embryo transfer (FET) cycles among women with adenomyosis and to develop internally validated prediction models using readily available clinical, laboratory, and imaging indicators.

**Methods:**

This retrospective cohort study included 673 FET cycles in women with imaging-confirmed adenomyosis treated at the Reproductive Center of Women and Children’s Hospital of Ningbo University between June 2018 and December 2024. Cycles ending in transfer failure or biochemical pregnancy were classified as non-clinical pregnancy, whereas cycles resulting in miscarriage or live birth were classified as clinical pregnancy. Among cycles achieving clinical pregnancy, outcomes were further categorized as miscarriage or live birth. Candidate predictors comprised demographic and infertility characteristics, anti-Müllerian hormone (AMH), pictorial blood loss assessment chart (PBAC) score, hemoglobin, carbohydrate antigen 125 (CA125), uterine volume, dysmenorrhea severity, adenomyosis type, endometrial-myometrial junctional zone involvement, and pretreatment modality. Separate multivariable logistic regression models were developed to predict clinical pregnancy and live birth. Model discrimination, calibration, and clinical usefulness were evaluated using the area under the receiver operating characteristic curve (AUC), bootstrap calibration, and decision curve analysis.

**Results:**

Of 673 cycles, 216 achieved clinical pregnancy (32.10%); 160 resulted in live birth (23.77%) and 56 in miscarriage. Independent predictors of clinical pregnancy included CA125, uterine volume, dysmenorrhea severity, adenomyosis type, JZ involvement, and pretreatment modality. Live birth was primarily predicted by CA125, uterine volume, JZ involvement, and pretreatment modality. Higher CA125, larger uterine volume, and JZ involvement were associated with miscarriage risk, whereas medical therapy was associated with improved live birth. These predictors were identified for risk prediction, the observed relationships should not be interpreted as causal treatment effects. The models showed good performance (AUC 0.830 for clinical pregnancy, 0.921 for live birth), with acceptable bootstrap calibration and net clinical benefit across a broad range of thresholds. Observed event rates increased across low, intermediate, and high predicted-probability strata.

**Conclusion:**

In adenomyosis patients undergoing FET, uterine volume, adenomyosis type, JZ involvement, dysmenorrhea severity, CA125, and pretreatment modality are key predictors of clinical pregnancy and live birth. The proposed models may support individualized risk stratification and clinical decision-making.

## Introduction

Adenomyosis is a complex, estrogen-dependent gynecological disorder defined by the presence of endometrial glands and stroma within the myometrium, accompanied by hyperplasia and hypertrophy of adjacent smooth muscle. These pathological changes contribute to myometrial thickening and an increased uterine volume (1, 2). Clinically, adenomyosis commonly presents with pelvic pain, abnormal uterine bleeding, and impaired fertility, while approximately 30% of affected women remain asymptomatic (3). Studies suggest that isolated adenomyosis is detected in approximately 10% of women presenting for infertility evaluation (4), with higher detection rates reported among patients with recurrent miscarriage, recurrent implantation failure, and advanced maternal age seeking pregnancy (5), which collectively supporting a close association between adenomyosis and impaired fertility.

The mechanisms by which adenomyosis compromises fertility are not yet fully elucidated, however, accumulating evidence suggests that it involves a combination of uterine structural and functional remodeling, impaired endometrial receptivity, and dysregulation of immune and molecular pathways. Structurally, adenomyosis is associated with thickening of the endometrial-myometrial junctional zone (JZ) and disruption of normal uterine peristalsis, which may impair sperm and embryo transport, alter intrauterine mechanical pressure, and ultimately hinder embryo implantation (6). At the molecular level, adenomyotic lesions and the surrounding eutopic endometrium exhibit a state of persistent chronic inflammation, characterized by increased proinflammatory cytokines. These changes are accompanied by aberrant expression of implantation-related factors, including HOXA10/HOXA11 and LIF, as well as progesterone resistance, leading to reduced endometrial receptivity and insufficient decidualization (7, 8). In addition, adenomyosis is associated with disturbances in the endometrial immune microenvironment, such as altered natural killer cell and macrophage populations (9) and dysregulation of cellular homeostasis processes, including autophagy (10), which may further increase the risk of implantation failure and early pregnancy loss. These structural and molecular alterations will result in decreased natural pregnancy rates, as well as reduced implantation rates, clinical pregnancy rates, and live birth rates in assisted reproductive technologies (ART), alongside increased miscarriage rates.

For patients with adenomyosis experiencing difficulties in achieving natural pregnancy or recurrent miscarriages, ART serves as an effective treatment option. However, embryo transfer failure not only signifies the loss of a single pregnancy opportunity but may also further exacerbate the negative impact of this condition on the patient’s physical and mental health. Multiple studies confirm that women with adenomyosis exhibit reduced clinical pregnancy and live birth rates in FET cycles, alongside significantly elevated spontaneous miscarriage rates (11, 12), indicating poorer overall reproductive outcomes. Concurrently, recurrent implantation failure (RIF) is closely associated with diminished fertility-related quality of life and increased risks of anxiety and depression, imposing substantial psychological burdens and financial pressures. This may even compromise patients’ willingness and adherence to continued treatment (13). Therefore, systematically evaluating pregnancy outcomes in FET cycles among patients with adenomyosis and identifying early factors affecting key outcomes like live birth holds significant clinical importance for optimizing assisted reproductive strategies, improving prognosis, and alleviating patients’ physical and psychological burdens. This study aims to analyze factors influencing FET outcomes in adenomyosis patients, providing evidence for individualized clinical decision-making.

## Methods

### Study design and subjects

**Figure 1:**
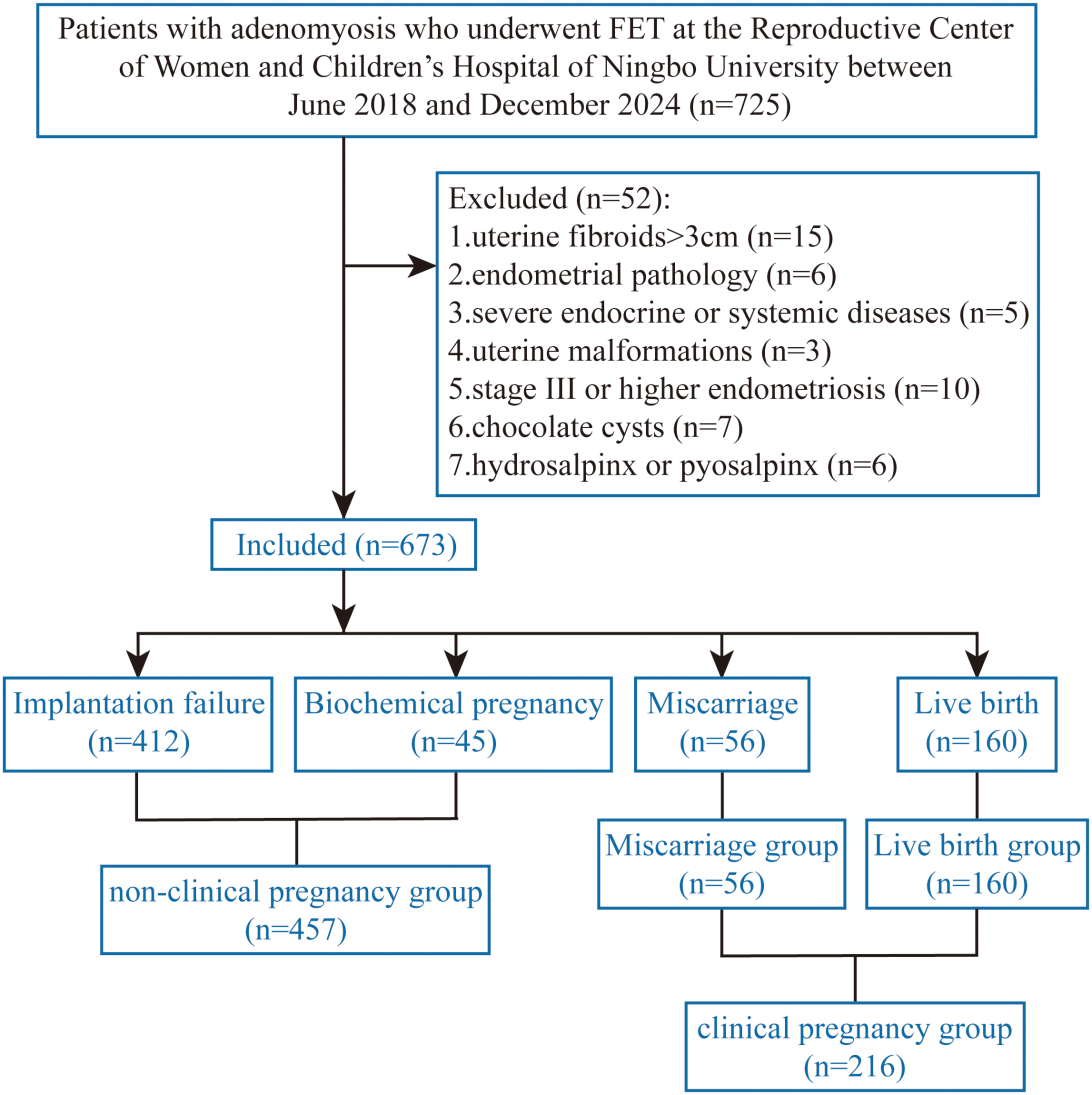
Flowchart.

This study is a single-center retrospective cohort study. Patients diagnosed with adenomyosis via ultrasound or MRI and undergoing FET at the Reproductive Center of Women and Children’s Hospital of Ningbo University between June 2018 and December 2024 were enrolled. Inclusion Criteria: Reproductive-age women with adenomyosis and normal ovarian reserve. Exclusion Criteria: Concurrent uterine fibroids>3cm; concurrent endometrial pathology; concurrent severe endocrine or systemic diseases; concurrent uterine malformations; concurrent stage III or higher endometriosis; concurrent chocolate cysts; concurrent hydrosalpinx or pyosalpinx. The flowchart is shown in **Figure 1**. A total of 673 FET cycles were included. The study protocol was reviewed and approved by the hospital’s ethics committee (Approval No.: NBFE-2025-KY-210). For each woman, we attempted to retrieve information on whether the corresponding ovarian stimulation cycle followed a freeze-all strategy or included a fresh embryo transfer prior to subsequent frozen transfers. Detailed ovarian stimulation parameters (protocol, gonadotropin type/total dose, number of oocytes retrieved, and number of embryos obtained) were not consistently available in a linkable format in our database and therefore could not be analyzed.

### Imaging evaluation of adenomyosis and definition of related variables

All patients underwent transvaginal three-dimensional ultrasound or pelvic MRI prior to transplantation, with images interpreted by experienced radiologists according to standardized criteria. Diagnosis of adenomyosis followed the revised Morphological Uterus Sonographic Assessment (MUSA) criteria (14), requiring the presence of ≥2 diagnostic features for inclusion. Key features included: Myometrial cysts; Hyperechogenic islands; Echogenic subendometrial lines and buds; Globular uterus; Asymmetrical myometrial thickening; Fan‐shaped shadowing; Translesional vascularity; Irregular junctional zone; Interrupted junctional zone. Adenomyosis types were classified as either focal (when normal myometrium surrounded >25% of the circumference of the lesion) or diffuse (if normal myometrium surrounded <25% or if it was difficult to differentiate focal from diffuse adenomyosis). Involvement of the junctional zone (JZ) is defined as lesion involvement or disruption of the endometrial-myometrial junctional zone. Uterine volume (V) is calculated using the ellipsoid formula by measuring longitudinal diameter (L), transverse diameter (W), and anteroposterior diameter (H):


V= (π/6) ×L×W×H

### Pretreatment modality and embryo transfer procedure

Pretreatment was categorized as no pretreatment, medical pretreatment, or surgical pretreatment prior to the index FET cycle. Medical pretreatment mainly consisted of depot gonadotropin-releasing hormone agonist (GnRH-a) injections (Leuprorelin Acetate Microspheres for Injection). The duration of GnRH-a pretreatment was individualized in routine practice, and the timing of embryo transfer was determined by experienced reproductive specialists based on overall clinical assessment and available indicators (e.g., symptom control), with FET initiated when the uterus was considered suitable for transfer. Surgical pretreatment referred to laparoscopic excision of adenomyosis lesions. To allow uterine recovery, embryo transfer was generally considered at least 6 months after surgery (**Supplementary Table 1**). All frozen embryo transfers in this study involved day-3 cleavage-stage (D3) embryos. All included FET cycles were derived from a freeze-all strategy; no fresh embryo transfers were performed in the study population.

### Outcome measures

This study defined two primary outcomes, with statistical units based on each transfer cycle. To avoid within-patient correlation, only the first eligible FET cycle per woman during the study period was included. Because longitudinal linkage of subsequent FET attempts beyond the index transfer was not feasible in our retrospective dataset, cumulative pregnancy and cumulative live birth rates across multiple frozen transfers could not be estimated. (i) Clinical Pregnancy Outcomes: Cycles labeled as “implantation failure” or “biochemical pregnancy” in “transfer outcomes” were defined as the non-clinical pregnancy group; live births and miscarriages were uniformly classified as the clinical pregnancy group. Biochemical pregnancy was defined as serum β-hCG≥7 mIU/mL without ultrasound confirmation. Clinical pregnancy was defined as ultrasound detection of an intrauterine gestational sac, embryo, or fetal heartbeat. (ii) Live Birth Outcomes: Among cycles achieving clinical pregnancy, delivery at ≥24 weeks gestation with viable fetal was defined as live birth. Pregnancy loss occurring before 24 weeks after clinical pregnancy was defined as miscarriage. Analysis of clinical pregnancy outcomes included all cycles meeting inclusion criteria; analysis of live birth outcomes included only cycles achieving clinical pregnancy.

### Statistical analysis

All statistical analyses were performed using R software version 4.3.1. Continuous variables were first tested for normality using the Shapiro-Wilk test. Variables approximating normal distribution were expressed as mean ± standard deviation (SD), and intergroup comparisons were performed using the independent samples t-test. For non-normally distributed variables, intergroup comparisons were conducted using the Mann-Whitney U test. Categorical variables were expressed as n (%), and intergroup comparisons were performed using the chi-square (χ²) test or Fisher’s exact test.

Univariate logistic regression analyses were conducted with “clinical pregnancy outcome” (non-clinical pregnancy = 0, clinical pregnancy = 1) and “live birth outcome” (miscarriage = 0, live birth = 1) as dependent variables. Odds ratios (ORs) with 95% confidence intervals (CIs) and two-tailed P values were reported. Candidate predictors were pre-specified based on clinical relevance and prior evidence, including age, BMI, duration of infertility, AMH, PBAC, HB, CA125, uterine volume, dysmenorrhea severity, adenomyosis type, JZ involvement, and pretreatment modality, and were entered simultaneously into multivariable logistic regression models. To construct parsimonious nomogram-based prediction models, we retained predictors that remained statistically significant after multivariable adjustment. Because the primary aim was prognostic model development rather than multiple hypothesis testing, no formal multiplicity correction (e.g., Bonferroni) was applied. Instead, P values were interpreted cautiously alongside model performance and internal validation. Separate multivariable models were established for clinical pregnancy and live birth, and adjusted ORs with 95% CIs were reported. For improved clinical interpretability, continuous predictors were rescaled to meaningful increments. CA125 was expressed per 10 U/mL increase, and uterine volume (recorded in mm^3^) was expressed per 10,000 mm^3^ (i.e., 10 cm^3^) increase. Corresponding ORs/aORs and 95% CIs were transformed accordingly.

Based on the multivariate models, the rms package was used to construct nomograms estimating individual outcome probabilities. Model discriminative power was evaluated via ROC curves and AUC. Bootstrap resampling (B = 500) generated calibration curves to assess consistency between predicted probabilities and actual outcomes. Decision curve analysis (DCA) compared the net benefit of “model-based decision”, “all interventions”, and “no intervention” at different threshold probabilities to evaluate the models’ potential clinical utility. Patients were stratified into three probability groups based on model-predicted probabilities: low, moderate, and high. Actual clinical pregnancy rates and live birth rates were compared across groups to evaluate the model’s prognostic stratification capability. All tests were two-sided, with P<0.05 indicating statistically significant differences.

## Results

### Baseline characteristics

A total of 673 FET cycles were included, comprising 457 non-clinical pregnancies and 216 clinical pregnancies (32.10%). Among the 216 cycles achieving clinical pregnancy, 160 resulted in live births (23.77%) and 56 in miscarriages (25.93%). All transfers involved day-3 (D3) embryos. All included cycles were frozen embryo transfers derived from a freeze-all strategy in the index stimulation cycle. No fresh embryo transfer was performed prior to the frozen transfers included in this analysis. Detailed distributions of adenomyosis phenotype/severity and pretreatment modality are provided in **Supplementary Table 2**. Within the clinical pregnancy analysis cohort, no statistically significant differences were observed between the non-clinical pregnancy group and the clinical pregnancy group in terms of age, BMI, duration of infertility, or AMH (all P>0.05, **Table 1**), indicating overall comparability of baseline characteristics between the two groups. Further stratification within the clinically pregnant cohort revealed no significant differences in age, BMI, duration of infertility, or AMH levels between the miscarriage and live birth groups (all P>0.05, **Table 2**).

**Table 1:** Comparison of baseline characteristics between non-clinical and clinical pregnancy groups.

Variables	Total (n=673)	Non-clinical pregnancy (n=457)	Clinical pregnancy (n=216)	P
Age^1^, years	34.38 ± 4.24	34.38 ± 4.34	34.38 ± 4.02	0.998
BMI^1^, kg/m^2^	23.09 ± 2.99	23.01 ± 3.03	23.25 ± 2.90	0.342
Infertility duration^1^, years	3.88 ± 2.33	3.88 ± 2.36	3.88 ± 2.27	0.849
AMH^1^, ng/mL	3.29 ± 2.69	3.25 ± 2.75	3.36 ± 2.58	0.286

^1^Mean ± SD.

**Table 2:** Comparison of baseline characteristics between miscarriage and live birth groups among clinically pregnant cycles.

Variables	Total (n=216)	Miscarriage (n=56)	Live birth (n=160)	P
Age^1^, years	34.38 ± 4.02	35.16 ± 4.59	34.10 ± 3.77	0.108
BMI^1^, kg/m^2^	23.25 ± 2.90	23.84 ± 3.59	23.04 ± 2.60	0.123
Infertility duration^1^, years	3.88 ± 2.27	4.02 ± 2.19	3.83 ± 2.30	0.619
AMH^1^, ng/mL	3.36 ± 2.58	2.85 ± 1.88	3.53 ± 2.77	0.250

^1^Mean ± SD.

### Univariate and multivariate regression analysis of clinical pregnancy outcomes

Univariate logistic regression analysis with “clinical pregnancy” as the outcome showed a mild negative correlation between PBAC score and clinical pregnancy (OR = 0.99, 95%CI 0.98-1.00, P = 0.049). For each 10 U/mL increase in CA125, the odds of clinical pregnancy decreased (OR = 0.73, 95% CI 0.63-0.84, P<0.001). Similarly, for each 10,000 mm^3^ (10 cm^3^) increase in uterine volume, the odds of clinical pregnancy decreased (OR = 0.64, 95% CI 0.58-0.72, P<0.001). Hemoglobin levels showed no significant association with clinical pregnancy. Compared to no dysmenorrhea, moderate-to-severe dysmenorrhea significantly reduced the likelihood of clinical pregnancy (OR = 0.44, 95%CI 0.29-0.68, P<0.001), while mild dysmenorrhea had no significant effect. Focal adenomyosis was associated with a higher likelihood of clinical pregnancy than diffuse adenomyosis (OR = 2.84, 95%CI 2.00-4.04, P<0.001). The highest clinical pregnancy rate was observed in those without JZ involvement, while the odds ratio for clinical pregnancy significantly decreased when JZ was involved (OR = 0.34, 95%CI 0.24-0.48, P<0.001). Pretreatment modalities also demonstrated a gradient effect: compared with no pretreatment, surgical intervention showed no significant change in odds ratio, whereas medical therapy significantly increased the clinical pregnancy rate (OR = 6.95, 95%CI 4.54-10.64, P<0.001) (**Table 3**).

**Table 3:** Univariate logistic regression analysis of factors associated with clinical pregnancy in adenomyosis patients undergoing FET.

Variables	Levels	OR (95% CI)	P
PBAC score	Per 1-unit increase	0.99 (0.98-1.00)	0.049
Hemoglobin, g/L	Per 1-unit increase	0.99 (0.94-1.04)	0.670
CA125, U/mL	Per 10 U/mL increase	0.73 (0.63-0.84)	<0.001
Uterine volume, mm^3^	Per 10,000 mm^3^ (10 cm^3^) increase	0.64 (0.58-0.72)	<0.001
Dysmenorrhea			<0.001
	None	1.00	
	Mild	1.20 (0.83-1.76)	
	Moderate-severe	0.44 (0.29-0.68)	
Adenomyosis type			<0.001
	Diffuse	1.00	
	Focal	2.84 (2.00-4.04)	
JZ involvement			<0.001
	No	1.00	
	Yes	0.34 (0.24-0.48)	
Pretreatment			<0.001
	No pretreatment	1.00	
	Surgery	1.16 (0.71-1.92)	
	Medication	6.95 (4.54-10.64)	

OR, odds ratio; CI, confidence interval.

After incorporating age, BMI, duration of infertility, AMH, PBAC, HB, CA125, uterine volume, dysmenorrhea severity, adenomyosis type, JZ involvement, and pretreatment modality into a multivariate logistic regression model, the effects of some indicators stabilized. CA125 remained an independent risk factor after adjustment. Per 10 U/mL increase, the adjusted OR was 0.84 (95% CI 0.72-0.99, P = 0.037). Increased uterine volume also remained independently associated with reduced clinical pregnancy. Per 10,000 mm^3^ (10 cm^3^) increase, the adjusted OR was 0.70 (95% CI 0.61-0.79, P<0.001). Regarding symptoms and structural factors, moderate-to-severe dysmenorrhea remained associated with lower clinical pregnancy rates (adjusted OR = 0.55, 95%CI 0.32-0.94, P = 0.029); Compared with diffuse adenomyosis, focal adenomyosis showed a higher odds ratio for clinical pregnancy (adjusted OR = 1.88, 95%CI 1.20-2.96, P = 0.006); involvement of JZ significantly reduced the probability of clinical pregnancy (adjusted OR = 0.41, 95%CI 0.27-0.61, P<0.001). Among pretreatment modalities, only medical therapy maintained significant benefit in the multivariable model (adjusted OR = 9.45, 95%CI 5.65-16.25, P<0.001), suggesting that medical intervention was independently associated with higher clinical pregnancy rates after controlling for other factors, whereas surgical therapy alone did not reach statistical significance (**Table 4**).

**Table 4:** Multivariable logistic regression analysis of factors associated with clinical pregnancy in adenomyosis patients undergoing FET.

Variables	Levels	Adjusted OR (95% CI)	P
Age, years	Per 1-unit increase	1.01 (0.96-1.06)	0.665
BMI, kg/m^2^	Per 1-unit increase	1.05 (0.98-1.12)	0.155
Infertility duration, years	Per 1-unit increase	0.98 (0.90-1.07)	0.610
AMH, ng/mL	Per 1-unit increase	0.98 (0.90-1.05)	0.577
PBAC score	Per 1-unit increase	0.99 (0.98-1.01)	0.294
Hemoglobin, g/L	Per 1-unit increase	0.95 (0.76-1.03)	0.626
CA125, U/mL	Per 10 U/mL increase	0.84 (0.72-0.99)	0.037
Uterine volume, mm^3^	Per 10,000 mm^3^ (10 cm^3^) increase	0.70 (0.61-0.79)	<0.001
Dysmenorrhea			0.044
	None	1.00	
	Mild	1.01 (0.64-1.61)	0.950
	Moderate-severe	0.55 (0.32-0.94)	0.029
Adenomyosis type			0.005
	Diffuse	1.00	
	Focal	1.88 (1.20-2.96)	0.006
JZ involvement			<0.001
	No	1.00	
	Yes	0.41 (0.27-0.61)	<0.001
Pretreatment			<0.001
	No pretreatment	1.00	
	Surgery	1.54 (0.85-2.73)	0.146
	Medication	9.45 (5.65-16.25)	<0.001

OR: odds ratio; CI: confidence interval.

Adjusted ORs with 95% CIs are shown. For categorical variables, the P value on the variable header row is the likelihood ratio test (overall effect); level-specific P values are Wald tests. P<0.001 is displayed as <0.001.

### Construction, performance evaluation, and probability stratification of a clinical pregnancy prediction model

**Figure 2:**
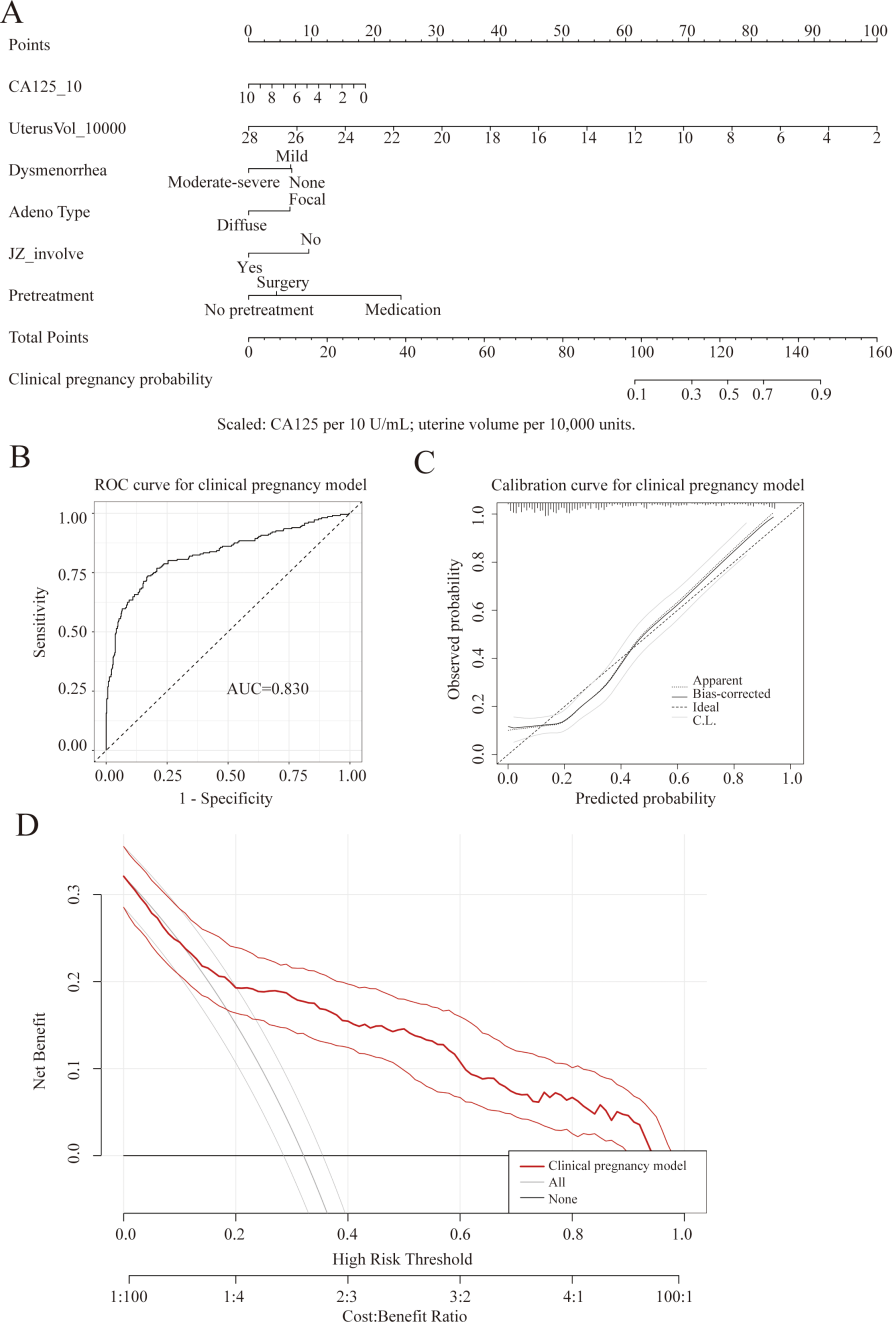
Clinical pregnancy prediction model in adenomyosis patients undergoing FET. **(A)** Nomogram for predicting the probability of clinical pregnancy based on CA125, uterine volume, dysmenorrhea severity, adenomyosis type, junctional zone (JZ) involvement, and pretreatment modality. **(B)** Receiver operating characteristic (ROC) curve of the model (AUC=0.830). **(C)** Calibration curve showing good agreement between predicted and observed clinical pregnancy rates. **(D)** Decision curve analysis demonstrating favorable net benefit across a range of clinically relevant threshold probabilities.

Based on the significant predictors identified in the multifactorial analysis (CA125, uterine volume, dysmenorrhea severity, adenomyosis type, JZ involvement, and pretreatment modality), a multifactorial logistic regression model was constructed with “clinical pregnancy” as the outcome. A nomogram was subsequently developed based on this model (**Figure 2A**). The nomogram assigns corresponding points to each variable, converting the cumulative total score into the probability of clinical pregnancy, thereby enabling visual risk estimation at the individual level.

The ROC curve showed that the model had an AUC of 0.830, indicating strong discriminatory ability between “non-clinical pregnancy” and “clinical pregnancy” (**Figure 2B**). In the calibration curve, the model’s predicted probabilities generally approached the ideal 45° line relative to observed clinical pregnancy rates, indicating good calibration (**Figure 2C**). Decision curve analysis further demonstrated that within commonly used clinical threshold probability ranges, this predictive model yields higher net benefit compared to strategies of “treating all as clinical pregnancies” or “treating all as non-clinical pregnancies”, suggesting its potential clinical utility (**Figure 2D**).

To evaluate the model’s performance in practical stratified management, the 673 cycles were divided into three groups based on the model-predicted clinical pregnancy probability: low (n=225), medium (n=224), and high (n=224). The median predicted probabilities (Q1-Q3) for the three groups were 0.083 (0.037-0.125), 0.231 (0.194-0.288), and 0.605 (0.484-0.818), respectively. The corresponding actual clinical pregnancy rates were 11.1%, 16.5%, and 68.8%, with statistically significant differences between groups (P<0.001) (**Table 5**). As the predicted probability stratification increased, the actual clinical pregnancy rate showed a marked gradient increase, indicating that this model not only possesses good statistical performance but also has clear clinical predictive probability stratification capability.

**Table 5:** Clinical pregnancy rates according to model-predicted probability strata.

Group	n	Predicted probability, median (Q1-Q3)	Observed event rate
Low	225	0.083 (0.037-0.125)	11.1%
Intermediate	224	0.231 (0.194-0.288)	16.5%
High	224	0.605 (0.484-0.818)	68.8%

Patients were stratified into low, medium, and high probability groups according to model-predicted clinical pregnancy probabilities.

### Univariate and multivariate regression analysis of live birth outcomes following clinical pregnancy

Among 216 cycles achieving clinical pregnancy, univariate logistic regression with “live birth” as the outcome revealed significant associations between CA125, uterine volume, adenomyosis type, JZ involvement, and pretreatment modality with live birth outcomes. In contrast, age, BMI, duration of infertility, AMH, PBAC, and HB showed no significant relationship with live birth rates. For each 10 U/mL increase in CA125, the odds of live birth decreased (OR = 0.28, 95% CI 0.18-0.45, P<0.001). Increased uterine volume was also associated with reduced live birth. Per 10,000 mm³ (10 cm³) increase, the OR was 0.49 (95% CI 0.39-0.63, P<0.001). Regarding structural and therapeutic factors, patients with focal adenomyosis had a higher live birth rate than those with diffuse adenomyosis (OR = 2.40, 95%CI 1.25-4.59, P = 0.009); involvement of JZ significantly reduced live birth rates (OR = 0.09, 95%CI 0.04-0.19, P<0.001). Compared with no pretreatment, both surgical and medical interventions were associated with higher live birth rates, with medical treatment showing the greatest advantage (OR = 5.40, 95%CI 2.50-11.64, P<0.001) (**Table 6**).

**Table 6:** Univariate logistic regression analysis of factors associated with live birth in adenomyosis patients undergoing FET.

Variables	Levels	OR (95% CI)	P
PBAC score	Per 1-unit increase	0.98 (0.95-1.01)	0.159
Hemoglobin, g/L	Per 1-unit increase	1.03 (0.72-1.46)	0.874
CA125, U/mL	Per 10 U/mL increase	0.28 (0.18-0.45)	<0.001
Uterine volume, mm^3^	Per 10,000 mm^3^ (10 cm^3^) increase	0.49 (0.39-0.63)	<0.001
Dysmenorrhea			0.165
	None	1.00	
	Mild	1.46 (0.72-2.96)	
	Moderate-severe	0.67 (0.30-1.47)	
Adenomyosis type			0.009
	Diffuse	1.00	
	Focal	2.40 (1.25-4.59)	
JZ involvement			<0.001
	No	1.00	
	Yes	0.09 (0.04-0.19)	
Pretreatment			<0.001
	No pretreatment	1.00	
	Surgery	2.87 (1.00-8.23)	
	Medication	5.40 (2.50-11.64)	

OR, odds ratio; CI, confidence interval.

In the multivariable model, after incorporating the same covariates as the clinical pregnancy model, several indicators still maintained independent predictive value for live birth outcomes. CA125 levels maintained a negative association after adjusting for confounders. Per 10 U/mL increase, the adjusted OR was 0.45 (95% CI 0.25-0.84, P = 0.012). The relationship between enlarged uterine volume and reduced live birth also remained significant. Per 10,000 mm^3^ (10 cm^3^) increase, the adjusted OR was 0.35 (95% CI 0.21-0.56, P<0.001). JZ involvement reduced the adjusted odds ratio for live birth to 0.11 (95%CI 0.04-0.31, P<0.001), indicating a more pronounced negative impact on pregnancy outcomes. Compared with no pretreatment, medication showed a significant positive association with live birth (adjusted OR = 22.13, 95%CI 6.12-105.49, P<0.001), whereas surgical intervention lost statistical significance after multivariable adjustment. Other variables did not demonstrate independent effects in the multivariable model (**Table 7**).

**Table 7:** Multivariable logistic regression analysis of factors associated with live birth in adenomyosis patients undergoing FET.

Variables	Levels	Adjusted OR (95% CI)	P
Age, years	Per 1-unit increase	0.95 (0.84-1.07)	0.402
BMI, kg/m^2^	Per 1-unit increase	0.93 (0.81-1.07)	0.303
Infertility duration, years	Per 1-unit increase	0.82 (0.65-1.03)	0.089
AMH, ng/mL	Per 1-unit increase	1.18 (0.94-1.50)	0.172
PBAC score	Per 1-unit increase	0.96 (0.91-1.01)	0.123
Hemoglobin, g/L	Per 1-unit increase	0.48 (0.22-1.02)	0.059
CA125, U/mL	Per 10 U/mL increase	0.45 (0.25-0.84)	0.012
Uterine volume, mm^3^	Per 10,000 mm^3^ (10 cm^3^) increase	0.35 (0.21-0.56)	<0.001
Dysmenorrhea			0.581
	None	1.00	
	Mild	1.13 (0.38-3.41)	0.820
	Moderate-severe	2.12 (0.51-9.88)	0.313
Adenomyosis type			0.265
	Diffuse	1.00	
	Focal	0.48 (0.12-1.74)	0.273
JZ involvement			<0.001
	No	1.00	
	Yes	0.11 (0.04-0.31)	<0.001
Pretreatment			<0.001
	No pretreatment	1.00	
	Surgery	2.39 (0.54-13.10)	0.274
	Medication	22.13 (6.12-105.49)	<0.001

OR, odds ratio; CI, confidence interval.

Adjusted ORs with 95% CIs are shown. For categorical variables, the P value on the variable header row is the likelihood ratio test (overall effect); level-specific P values are Wald tests. P<0.001 is displayed as <0.001.

### Construction, performance evaluation, and probability stratification of the live birth prediction model

**Figure 3:**
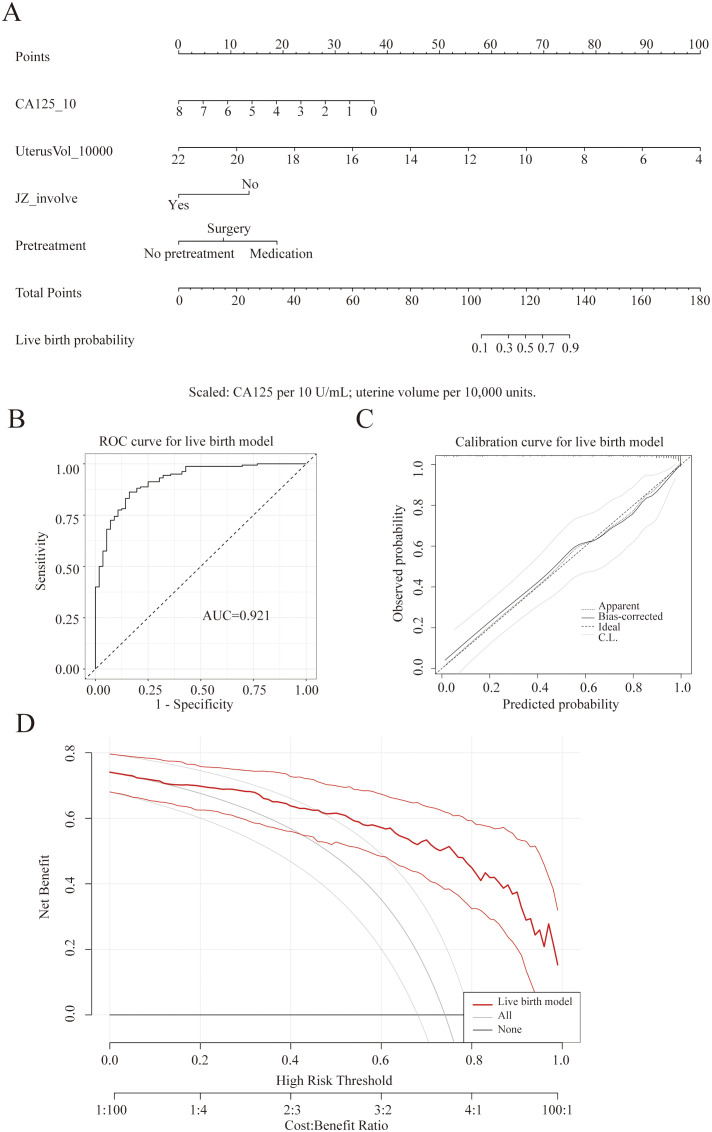
Live birth prediction model among clinical pregnancy cycles. **(A)** Nomogram for predicting live birth probability based on CA125, uterine volume, JZ involvement, and pretreatment modality. **(B)** Receiver operating characteristic (ROC) curve of the model (AUC=0.921). **(C)** Calibration curve showing good concordance between predicted and observed live birth outcomes. **(D)** Decision curve analysis indicating superior net clinical benefit over “all intervention” and “no intervention” strategies.

A live birth prediction model for clinical pregnancies was constructed based on four independent predictors: CA125, uterine volume, JZ involvement, and pretreatment modality. This model therefore predicts the probability of live birth conditional on having achieved clinical pregnancy, and is intended for early-pregnancy prognostic stratification rather than pre-transfer estimation of overall live birth probability. A corresponding nomogram was generated (**Figure 3A**). Cumulative scores enable intuitive estimation of the probability of achieving a live birth for each clinical pregnancy cycle, providing quantitative evidence for early pregnancy prognosis assessment and follow-up strategies. The ROC curve revealed an AUC of 0.921 (**Figure 3B**), indicating strong discriminatory ability between “miscarriage” and “live birth”. To examine potential overfitting, we further evaluated model optimism using bootstrap internal validation; the optimism-corrected performance metrics are reported in **Supplementary Table 3**. Calibration curves demonstrated overall good fit between predicted probabilities and actual live birth rates, with only minor deviations observed in the high-prediction probability range (**Figure 3C**). Decision curve analysis suggests that within a broad range of threshold probabilities, this live birth prediction model yields greater net clinical benefit than either “all intervention” or “no intervention” strategies, supporting its potential value as a clinical decision-making tool (**Figure 3D**).

Similarly, based on model predictions of live birth probability, 216 clinical pregnancy cycles were categorized into low, medium, and high probability groups (72 cases each). The median predicted probabilities (Q1-Q3) for the low, medium, and high probability groups were 0.335 (0.109-0.570), 0.903 (0.845-0.933), and 0.987 (0.975-0.994), respectively. The corresponding actual live birth rates were 34.7%, 88.9%, and 98.6%, with significant differences between groups (P<0.001) (**Table 8**). While the observed live birth rate increased markedly across the three predicted-probability strata, the very high predicted probabilities in the highest stratum should be interpreted with caution given potential optimism in internally developed models, underscoring the need for external validation.

**Table 8:** Live birth rates according to model-predicted probability strata.

Group	n	Predicted probability, median (Q1-Q3)	Observed event rate
Low	72	0.335 (0.109-0.570)	34.7%
Intermediate	72	0.903 (0.845-0.933)	88.9%
High	72	0.987 (0.975-0.994)	98.6%

Analysis was restricted to cycles achieving clinical pregnancy (n = 216). Patients were stratified into low, medium, and high probability groups according to model-predicted live birth probabilities.

## Discussion

This study, based on a large single-center cohort of patients with adenomyosis undergoing FET cycles, defined “implantation failure + biochemical pregnancy” as non-clinical pregnancy group and “miscarriage + live birth” as clinical pregnancy group. Among cycles achieving clinical pregnancy, miscarriages were categorized as miscarriage group and live births as live birth group. Multivariate prediction models were constructed separately for clinical pregnancy and live birth. Results showed that uterine volume, involvement of the endometrial-myometrial junctional zone (JZ), and pretreatment modality were stable independent predictors in both models. Dysmenorrhea severity and adenomyosis type were primarily associated with achieving clinical pregnancy, while serum CA125 demonstrated stronger association with live birth outcomes. The decision curves constructed based on these variables demonstrated good discrimination (AUC of approximately 0.830 for the clinical pregnancy model and approximately 0.921 for the live birth model) and calibration. Decision curves and probability stratification further suggested that the models possess certain potential for clinical application.

In this adenomyosis-only cohort, the clinical pregnancy rate after FET was 32.10%. Because the present study did not include a non-adenomyosis control group, we did not perform between-group comparisons, and the observed pregnancy outcomes should be interpreted as descriptive estimates within women with adenomyosis. Comparisons with other infertility populations should rely on external literature rather than inference from our dataset. Uterine structural remodeling and involvement of the uterine consistently correlated closely with both clinical pregnancy outcomes and live birth outcomes in this study, suggesting these may represent key pathological mechanisms through which adenomyosis impacts reproductive prognosis. On one hand, increased uterine volume was significantly associated with reduced clinical pregnancy and live birth rates in both models, while JZ involvement remained a strong adverse factor (OR approximately 0.3-0.4) in multivariate analysis. Previous studies have indicated that adenomyosis causes thickening, disruption, and abnormal rhythmic contractions of the JZ, impairing sperm and embryo transport as well as uterine pressure, thereby reducing endometrial receptivity during the implantation window (6). More critically, JZ dysfunction and impaired local blood perfusion may persist during early pregnancy, increasing the risk of miscarriage and placental complications (15). Although we did not longitudinally assess JZ changes during early pregnancy, prior work suggests that impaired remodeling of the inner myometrium/junctional zone before conception may predispose to defective deep placentation and adverse pregnancy outcomes (16–18). Recent reviews further support the role of junctional zone structure/function in adenomyosis-related infertility and obstetric complications (6). Our findings directly link these structural and functional abnormalities to two key clinical outcomes “achieving clinical pregnancy” and “ultimately delivering a live birth.” This indicates that uterine volume and JZ involvement should be incorporated as critical considerations in risk assessment and intervention decisions for assisted reproductive management in adenomyosis patients, rather than merely serving as diagnostic imaging information. Clinical and laboratory indicators reflecting disease activity and burden play distinct roles across different outcome stages. Moderate-to-severe dysmenorrhea significantly reduced the probability of clinical pregnancy in both univariate and multivariate models using clinical pregnancy outcomes as endpoints, but its independent impact on live birth outcomes was weaker. This suggests pain primarily reflects lesion activity and abnormal contractions directly interfering with the implantation phase. Once pregnancy is established, its maintenance through to live birth relies more heavily on the local and systemic inflammatory, vascular, and immune environments within the uterus (19, 20). Serum CA125 levels showed significant negative correlations with both clinical pregnancy and live birth in this study, particularly emerging as an independent risk factor in the live birth model. This aligns with CA125’s role as a marker of adenomyosis lesion extent and inflammatory burden (21, 22). Although some studies reported no significant association between CA125 levels prior to hormone replacement cycle FET and outcomes in adenomyosis patients (23), these studies often failed to distinguish between different outcome stages and did not adequately account for important confounding factors such as JZ involvement and pretreatment modality. This study suggests that in adenomyosis patients, the relationship between CA125 and uterine volume, JZ involvement, and symptom severity should be interpreted holistically. CA125 should be viewed as a component within the “disease burden-uterine environment-pregnancy outcome” chain rather than a standalone linear predictor.

Pretreatment modality was strongly associated with both clinical pregnancy and live birth in this study, however, these findings must be interpreted in the context of treatment indications. In routine practice, treatment selection (particularly surgery) is typically driven by baseline disease severity, symptom burden, and prior treatment failures, making confounding by indication likely. To evaluate the impact of this potential bias, we conducted sensitivity analyses excluding pretreatment modality from the models; discrimination remained acceptable, supporting the robustness of the non-pretreatment predictors (**Supplementary Table 3**). Consistent with clinical practice, fertility-sparing surgery for adenomyosis is often considered a “last resort,” usually reserved for patients with markedly enlarged uterine volume, severe dysmenorrhea, multiple failed embryo transfers, or symptoms significantly impairing quality of life (24, 25). Therefore, the surgical group in our cohort likely represented patients with a greater underlying disease burden and a more complex treatment history. Accordingly, the lower clinical pregnancy and live birth rates observed in the surgical group compared with the medical group are more plausibly attributable to baseline severity and prior treatment failures rather than indicating that surgery itself is “ineffective” or “harmful.” Taken together, the associations between pretreatment modality and pregnancy outcomes in our cohort should be interpreted as observational correlations rather than causal effects. Moreover, GnRH-a pretreatment was individualized and the decision to proceed with embryo transfer was made once the uterus was considered suitable for transfer, which may introduce additional heterogeneity and selection effects. Future prospective studies with standardized pretreatment protocols and explicit criteria for treatment selection are warranted.

Finally, the two predictive models developed in this study, together with their nomograms, ROC curves, calibration plots, decision curves, and probability stratification, provide intuitive tools for clinical risk assessment. In the clinical pregnancy model, the observed clinical pregnancy rate increased from approximately 11% to nearly 70% across the three predicted-probability strata (lowest to highest). In the live birth model, the corresponding live birth rate rose from about 35% to nearly 100%, demonstrating a clear outcome gradient that supports the use of these models for prognostic stratification. In practice, patients classified as low probability may benefit from closer evaluation and shared decision-making regarding pre-transfer optimization (e.g., timing of transfer and broader optimization of the uterine environment), however, whether any specific intensified intervention improves outcomes cannot be inferred from this retrospective analysis and requires prospective confirmation. Conversely, for patients with higher predicted probabilities, unnecessary escalation of interventions may be avoided, potentially reducing treatment burden.

The strengths of this study include a uniformly diagnosed adenomyosis cohort based on imaging, inclusion of FET cycles only, integration of symptom, laboratory, imaging, and pretreatment variables, and comprehensive model evaluation using discrimination, calibration, decision curves, and risk stratification. Nevertheless, several limitations should be acknowledged. First, the single-center retrospective design is susceptible to selection and information biases. Second, the absence of a non-adenomyosis control group precludes direct comparative conclusions regarding whether pregnancy rates are lower than those of other infertility populations, and residual within-patient correlation may remain, potentially leading to underestimated standard errors. In addition, assessments of JZ involvement and adenomyosis type lacked standardized quantitative criteria. Key determinants of FET outcomes—such as blastocyst status, endometrial thickness at transfer, and endometrial preparation protocols—were not consistently recorded and therefore could not be incorporated, which may introduce residual confounding. Furthermore, our retrospective database did not allow reliable longitudinal linkage of subsequent FET attempts beyond the index transfer, therefore, cumulative clinical pregnancy and cumulative live birth rates across multiple frozen transfers could not be estimated. Detailed ovarian stimulation-cycle characteristics (e.g., stimulation protocol, number of oocytes retrieved, and number of embryos obtained) were also not consistently recorded in a linkable format. Although we reported whether a freeze-all strategy or a preceding fresh transfer occurred, the absence of comprehensive stimulation parameters may limit generalizability and introduces potential residual confounding. Notably, all transfers in this cohort used cleavage-stage (D3) embryos, partially reducing heterogeneity related to embryo stage. Moreover, the live birth model was developed among cycles achieving clinical pregnancy and thus is intended for prognostic stratification conditional on clinical pregnancy rather than comprehensive pre-transfer estimation of overall live birth probability. Treatment regimens were heterogeneous, and the models have undergone internal validation only; external validation in multicenter prospective cohorts (ideally using a unified imaging grading system and incorporating embryo-related variables and pregnancy complications) is required before broader implementation. Future studies evaluating whether risk-stratified management improves real-world outcomes will further clarify the clinical utility of these models in adenomyosis patients.

## Data Availability

The original contributions presented in the study are included in the article/**Supplementary Material**. Further inquiries can be directed to the corresponding authors.

## References

[1] Kho KA Chen JS Halvorson LM . Diagnosis, evaluation, and treatment of adenomyosis. Jama. (2021) 326:177–8. doi: 10.1001/jama.2020.26436, PMID: 34255015

[2] Wang R Xu S Cui Q Chen X Wang X Liu J . Single-cell RNA sequencing identifies the prolactin receptor as a therapeutic target in adenomyosis. Signal Transduct Target Ther. (2025) 10:258. doi: 10.1038/s41392-025-02339-z, PMID: 40804233 PMC12350698

[3] Gordts S Grimbizis G Campo R . Symptoms and classification of uterine adenomyosis, including the place of hysteroscopy in diagnosis. Fertil Steril. (2018) 109:380–8.e1. doi: 10.1016/j.fertnstert.2018.01.006, PMID: 29566850

[4] Mishra I Melo P Easter C Sephton V Dhillon-Smith R Coomarasamy A . Prevalence of adenomyosis in women with subfertility: systematic review and meta-analysis. Ultrasound Obstet Gynecol. (2023) 62:23–41. doi: 10.1002/uog.26159, PMID: 36647238

[5] Puente JM Fabris A Patel J Patel A Cerrillo M Requena A . Adenomyosis in infertile women: prevalence and the role of 3D ultrasound as a marker of severity of the disease. Reprod Biol Endocrinol. (2016) 14:60. doi: 10.1186/s12958-016-0185-6, PMID: 27645154 PMC5029059

[6] Wang S Duan H . The role of the junctional zone in the management of adenomyosis with infertility. Front Endocrinol (Lausanne). (2023) 14:1246819. doi: 10.3389/fendo.2023.1246819, PMID: 37886646 PMC10598341

[7] Bulun SE Yildiz S Adli M Wei JJ . Adenomyosis pathogenesis: insights from next-generation sequencing. Hum Reprod Update. (2021) 27:1086–97. doi: 10.1093/humupd/dmab017, PMID: 34131719 PMC8543024

[8] Hiraoka T Hirota Y Osuga Y . How does adenomyosis impact endometrial receptivity? An updated systematic review of clinical and molecular insights. F&S Rev. (2023) 4:15–25. doi: 10.1016/j.xfnr.2022.11.004, PMID: 41768852

[9] Shi J Xu Q Yu S Zhang T . Perturbations of the endometrial immune microenvironment in endometriosis and adenomyosis: their impact on reproduction and pregnancy. Semin Immunopathol. (2025) 47:16. doi: 10.1007/s00281-025-01040-1, PMID: 39966111 PMC11835911

[10] Vervier J Squatrito M Nisolle M Henry L Munaut C . Controversial roles of autophagy in adenomyosis and its implications for fertility outcomes-A systematic review. J Clin Med. (2024) 13. doi: 10.3390/jcm13247501, PMID: 39768424 PMC11676161

[11] Liang T Zhang W Pan N Han B Li R Ma C . Reproductive outcomes of *in vitro* fertilization and fresh embryo transfer in infertile women with adenomyosis: A retrospective cohort study. Front Endocrinol (Lausanne). (2022) 13:865358. doi: 10.3389/fendo.2022.865358, PMID: 35966061 PMC9372912

[12] Cozzolino M Tartaglia S Pellegrini L Troiano G Rizzo G Petraglia F . The effect of uterine adenomyosis on IVF outcomes: a systematic review and meta-analysis. Reprod Sci. (2022) 29:3177–93. doi: 10.1007/s43032-021-00818-6, PMID: 34981458

[13] Guo YB Tang B Zhang L Wu X Huang ZH . Anxiety and depression in recurrent implantation failure after frozen-thawed embryo transfer and efficacy of endometrial receptivity testing. World J Psychiatry. (2025) 15:109175. doi: 10.5498/wjp.v15.i9.109175, PMID: 40933169 PMC12417924

[14] Harmsen MJ Van Den Bosch T De Leeuw RA Dueholm M Exacoustos C Valentin L . Consensus on revised definitions of Morphological Uterus Sonographic Assessment (MUSA) features of adenomyosis: results of modified Delphi procedure. Ultrasound Obstet Gynecol. (2022) 60:118–31. doi: 10.1002/uog.24786, PMID: 34587658 PMC9328356

[15] Horton J Sterrenburg M Lane S Maheshwari A Li TC Cheong Y . Reproductive, obstetric, and perinatal outcomes of women with adenomyosis and endometriosis: a systematic review and meta-analysis. Hum Reprod Update. (2019) 25:592–632. doi: 10.1093/humupd/dmz012, PMID: 31318420

[16] Brosens JJ Pijnenborg R Brosens IA . The myometrial junctional zone spiral arteries in normal and abnormal pregnancies: a review of the literature. Am J Obstet Gynecol. (2002) 187:1416–23. doi: 10.1067/mob.2002.127305, PMID: 12439541

[17] Brosens I Derwig I Brosens J Fusi L Benagiano G Pijnenborg R . The enigmatic uterine junctional zone: the missing link between reproductive disorders and major obstetrical disorders? Hum Reprod. (2010) 25:569–74. doi: 10.1093/humrep/dep474, PMID: 20085913

[18] Vercellini P Viganò P Bandini V Buggio L Berlanda N Somigliana E . Association of endometriosis and adenomyosis with pregnancy and infertility. Fertil Steril. (2023) 119:727–40. doi: 10.1016/j.fertnstert.2023.03.018, PMID: 36948440

[19] Abu-Raya B Michalski C Sadarangani M Lavoie PM . Maternal immunological adaptation during normal pregnancy. Front Immunol. (2020) 11:575197. doi: 10.3389/fimmu.2020.575197, PMID: 33133091 PMC7579415

[20] Menzies FM . The placenta as an immunological environment. Br J BioMed Sci. (2025) 82:14910. doi: 10.3389/bjbs.2025.14910, PMID: 41341663 PMC12669068

[21] Su B Huang JR Wang H Fang CL He HN Tang W . Combined magnetic resonance imaging with serum CA125 for dysmenorrhea in adenomyosis. Sci Rep. (2025) 15:42317. doi: 10.1038/s41598-025-26412-3, PMID: 41309741 PMC12660971

[22] Nishida H Takehara K Onodera T Watanabe S Takasaki K Takahashi Y . Sequential therapy of dienogest following relugolix for adenomyosis and impact on symptoms and serum CA125 levels: a case series. BMC Womens Health. (2025) 25:150. doi: 10.1186/s12905-025-03681-8, PMID: 40158154 PMC11954200

[23] Huang L Li Y Chen M Wang Z Zhou C . Serum levels of cancer antigen 125 before hormone replacement therapy are not associated with clinical outcome of frozen embryo transfer in women with adenomyosis. J Int Med Res. (2021) 49:3000605211005878. doi: 10.1177/03000605211005878, PMID: 33887984 PMC8072100

[24] Kim H Frisch EH Falcone T . From diagnosis to fertility: optimizing treatment of adenomyosis for reproductive health. J Clin Med. (2024) 13. doi: 10.3390/jcm13164926, PMID: 39201068 PMC11355825

[25] Hamaguchi F Kitawaki Y Ohara T Miyamoto T Okunomiya A Sunada M . Pregnancy outcomes with and without adenomyomectomy in infertile patients with adenomyosis: A single-center retrospective study. J Obstet Gynaecol Res. (2025) 51:e70149. doi: 10.1111/jog.70149, PMID: 41320195 PMC12665454

