# Supplementary material for: Impact of adenomyosis on pregnancy outcomes following frozen embryo transfer: development and validation of a single-center clinical predictive model
Source: Front Endocrinol (Lausanne). 2026 Mar 5;17:1770158. doi: 10.3389/fendo.2026.1770158 (PMC12999379; doi:10.3389/fendo.2026.1770158)
Supplement: Supplementary file 1 [file Table1.docx]

Table S1. Details of pretreatment modalities

| Item | Medication (GnRH-a, Leuprorelin Acetate Microspheres for Injection) | Surgery (laparoscopic excision) |
| --- | --- | --- |
| Definition | Depot GnRH-a injections prior to the index FET; duration individualized until uterus deemed suitable for transfer | Laparoscopic excision of adenomyosis lesion(s) (adenomyomectomy/excision) |
| No. of cycles, n (%) | 132 (19.61%) | 101 (15.01%) |
| Key timing | During routine practice, transfer initiated when clinically deemed suitable for transfer | Embryo transfer generally considered ≥6 months after surgery |
| Note | Decision to proceed with FET made by experienced reproductive specialists based on clinical/imaging assessment | Interval intended to allow uterine recovery |

Table S2A. By clinical pregnancy outcome (n=673 cycles)

| Variables | Total  (n=673) | Non-clinical  Pregnancy  (n=457) | Clinical  Pregnancy  (n=216) | P |
| --- | --- | --- | --- | --- |
| Dysmenorrhea, n (%) |  |  |  | <0.001 |
| None | 238 (35.36%) | 153 (33.48%) | 85 (39.35%) |  |
| Mild | 222 (32.99%) | 133 (29.10%) | 89 (41.20%) |  |
| Moderate–severe | 213 (31.65%) | 171 (37.42%) | 42 (19.44%) |  |
| Adenomyosis type, n (%) |  |  |  | <0.001 |
| Diffuse | 295 (43.83%) | 236 (51.64%) | 59 (27.31%) |  |
| Focal | 378 (56.17%) | 221 (48.36%) | 157 (72.69%) |  |
| JZ involvement, n (%) |  |  |  | <0.001 |
| No | 304 (45.17%) | 168 (36.76%) | 136 (62.96%) |  |
| Yes | 369 (54.83%) | 289 (63.24%) | 80 (37.04%) |  |
| Pretreatment modality, n (%) |  |  |  | <0.001 |
| No pretreatment | 440 (65.38%) | 339 (74.18%) | 101 (46.76%) |  |
| Medication (GnRH-a) | 132 (19.61%) | 43 (9.41%) | 89 (41.20%) |  |
| Surgery (laparoscopic excision) | 101 (15.01%) | 75 (16.41%) | 26 (12.04%) |  |
| Uterine volume, mm^3^ (mean ± SD) | 78042.47± 27632.82 | 83835.98± 28956.16 | 65784.89± 19646.99 | <0.001 |
| CA125, U/mL (mean ± SD) | 21.65 ±15.13 | 23.44 ±16.90 | 17.88 ±9.43 | <0.001 |

Table S2B. By live birth outcome among clinical pregnancies (n=216 cycles)

| Variables | Total  (n=216) | Miscarriage (n=56) | Live birth  (n=160) | P |
| --- | --- | --- | --- | --- |
| Dysmenorrhea, n (%) |  |  |  | 0.161 |
| None | 85 (39.35%) | 23 (41.07%) | 62 (38.75%) |  |
| Mild | 89 (41.20%) | 18 (32.14%) | 71 (44.38%) |  |
| Moderate–severe | 42 (19.44%) | 15 (26.79%) | 27 (16.88%) |  |
| Adenomyosis type, n (%) |  |  |  | 0.009 |
| Diffuse | 59 (27.31%) | 23 (41.07%) | 36 (22.50%) |  |
| Focal | 157 (72.69%) | 33 (58.93%) | 124 (77.50%) |  |
| JZ involvement, n (%) |  |  |  | <0.001 |
| No | 136 (62.96%) | 13 (23.21%) | 123 (76.88%) |  |
| Yes | 80 (37.04%) | 43 (76.79%) | 37 (23.12%) |  |
| Pretreatment modality, n (%) |  |  |  | <0.001 |
| No pretreatment | 101 (46.76%) | 41 (73.21%) | 60 (37.50%) |  |
| Medication (GnRH-a) | 89 (41.20%) | 10 (17.86%) | 79 (49.38%) |  |
| Surgery (laparoscopic excision) | 26 (12.04%) | 5 (8.93%) | 21 (13.12%) |  |
| Uterine volume, mm^3^ (mean ± SD) | 65784.89± 19646.99 | 80330.70± 28755.28 | 60693.86± 11633.22 | <0.001 |
| CA125, U/mL (mean ± SD) | 17.88 ± 9.43 | 24.79 ± 12.96 | 15.46 ± 6.29 | <0.001 |

Table S3. Sensitivity analyses addressing pretreatment modality inclusion and model optimism (bootstrap internal validation).

| Model / Endpoint | Predictors included | AUC (apparent) | AUC (optimism-corrected) | Brier (apparent) | Brier (optimism-corrected) |
| --- | --- | --- | --- | --- | --- |
| Model 1: Clinical pregnancy (all cycles) | CA125, uterine volume, dysmenorrhea severity, adenomyosis type, JZ involvement, pre**treatment modality** | 0.830 | 0.824 | 0.141 | 0.144 |
| Model 1 (sensitivity): Clinical pregnancy | Same as above **excluding pretreatment modality** | 0.787 | 0.781 | 0.164 | 0.167 |
| Model 2: Live birth **among clinical pregnancies** | CA125, uterine volume, JZ involvement, pre**treatment modality** | 0.921 | 0.907 | 0.093 | 0.103 |
| Model 2 (sensitivity): Live birth among clinical pregnancies | Same as above **excluding pretreatment modality** | 0.879 | 0.873 | 0.115 | 0.122 |
| Model 3 (supplementary): Overall live birth (all cycles) | CA125, uterine volume, dysmenorrhea severity, adenomyosis type, JZ involvement, pre**treatment modality** | 0.929 | 0.924 | 0.082 | 0.086 |
| Model 3 (sensitivity): Overall live birth | Same as above **excluding pretreatment modality** | 0.880 | 0.875 | 0.108 | 0.111 |

AUC: area under the ROC curve. Optimism-corrected estimates were obtained via bootstrap resampling (B=500).
